# Rare *Proteus mirabilis* Aortic Valve Infective Endocarditis without a Urinary Tract Infection

**DOI:** 10.1155/2022/7569148

**Published:** 2022-12-31

**Authors:** Sarah Gorn, Nicholas Talley, Austin Mitchell, Alejandro Pineda

**Affiliations:** Midwestern University, Mountain Vista Medical Center, Mesa, AZ, USA

## Abstract

*Proteus mirabilis* infective endocarditis is a rare disease with only 17 reported cases. It is typically associated with urinary tract infections (UTIs), staghorn calculi, and/or asymptomatic bacteriuria. We present a case of a 73-year-old male who presented with positive blood cultures for *Proteus mirabilis* but with a negative urinalysis and urine culture. He presented with acute renal failure and required hemodialysis. TTE was remarkable for a 30% ejection fraction, and no vegetations were visualized. TEE demonstrated a small vegetation on the left aortic valve. The initial urine culture remained negative throughout his hospitalization. He was treated with IV antibiotics and discharged without hemodialysis.

## 1. Introduction

Infective endocarditis (IE) is a rare disease with significant mortality. It is most commonly caused by Gram-positive bacteria; however, Gram-negative bacteria have been known to cause IE as well. IE secondary to *P. mirabilis*, however, is extremely rare, with only 17 known reported cases [1, 2]. *P. mirabilis* is typically associated with urinary tract infections, specifically in elderly patients, with risk factors such as catheters, urological anatomic anomalies, or diabetes. [1]. None of these conditions were present in our patient. The virulence of *P. mirabilis* infection can lead to bacteremia and rarely progresses to IE. We present a rare case of native valve IE due to *P. mirabilis* which is the first known occurrence without a concurrent UTI or asymptomatic bacteriuria.

## 2. Case Report

**Figure 1:**
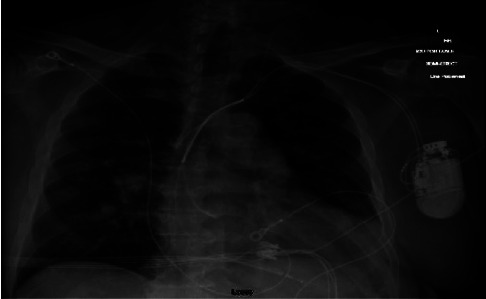
Chest X-ray.

**Figure 2:**
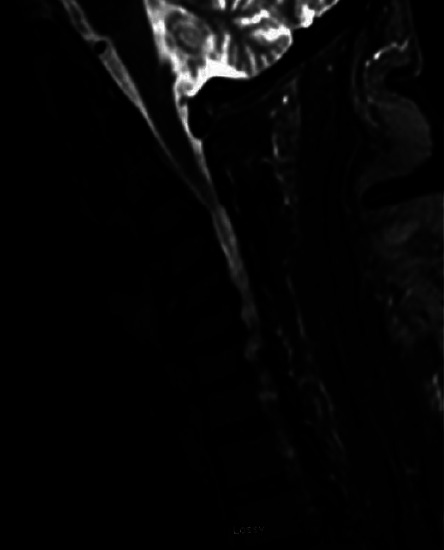
MRI cervical spine.

**Figure 3:**
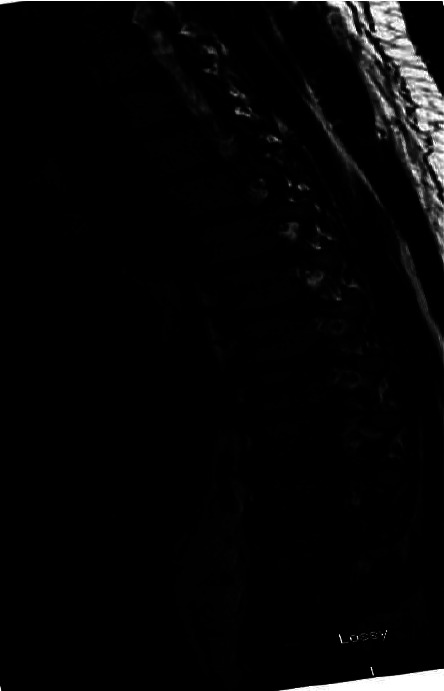
MRI thoracic spine.

**Figure 4:**
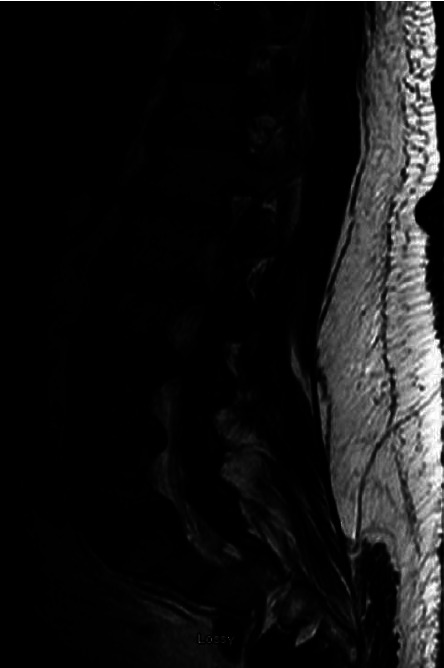
MRI lumbar spine.

**Figure 5:**
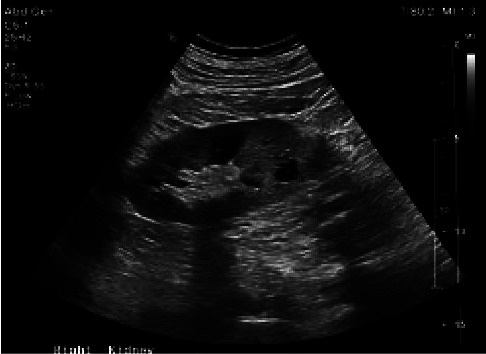
Renal US right kidney.

**Figure 6:**
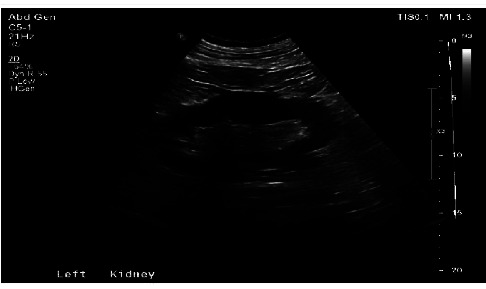
Renal US left kidney.

**Figure 7:**
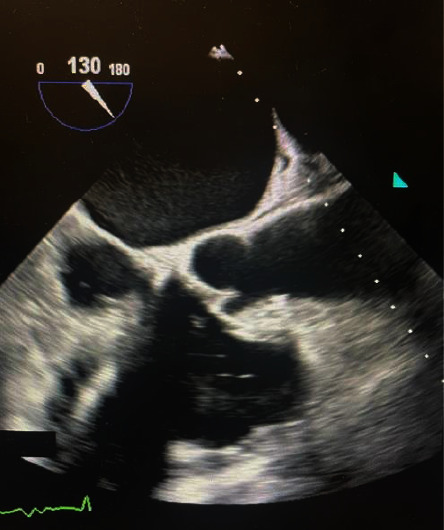
TEE of aortic valve vegetation 1.

**Figure 8:**
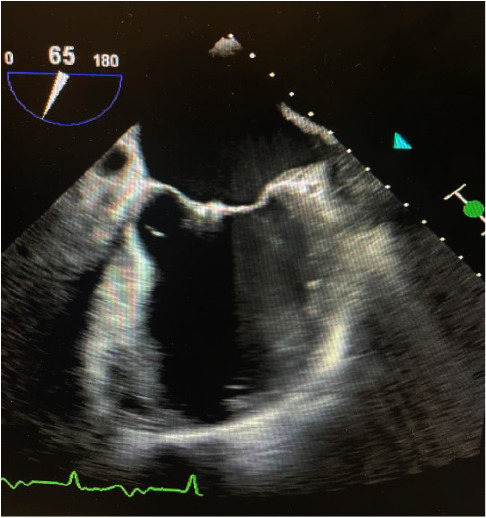
TEE of aortic valve vegetation 2.

A 73-year-old African-American male with a past medical history of coronary artery disease status after percutaneous coronary intervention, chronic systolic heart failure with an automatic implantable cardioverter defibrillator (AICD) placed 9 months prior, atrial fibrillation, and a mitral clip presented with altered mentation and worsening bilateral flank pain for three weeks duration with associated decreased urine output. His presenting blood pressure was 90/70. Heart rate, respiratory rate, and oxygen saturation was unremarkable. Physical examination was notable for somnolence. Labs were significant for WBC 9.5 (normal range: 4.0–11.0), potassium 5.5 (normal range 3.5–5.1), creatinine 6.4 (normal range: 0.60–1.30), and BUN 120 (normal range: 7–18). Blood cultures on admission were positive for *Proteus mirabilis* and remained positive on repeat blood cultures drawn 48 hours later. The urinalysis and the urine culture were unremarkable. A chest radiograph revealed mild pneumonitis in the right lung (Figure 1). Magnetic resonance imaging and computed tomography of the cervical, thoracic, and lumbar spine were negative for infection or abscess (Figures 2–4). A nuclear white blood cell scan showed increased uptake in the bilateral lungs. Renal ultrasound was remarkable for simple cysts in both the kidneys (Figures 5 and 6). A transthoracic echocardiogram revealed an ejection fraction of 35–40% with mild aortic regurgitation. A transesophageal echocardiogram revealed a small vegetation of the aortic valve (Figures 7 and 8). He was treated with meropenem 1g q12 h for one day, initially for empiric therapy. Based on identification of *Proteus mirabilis*, he was deescalated to ceftriaxone 2g qd for 6 days. On day 6, a vegetation of the aortic valve was revealed, and ciprofloxacin 400 mg q12 h was added for dual coverage. The patient remained on ceftriaxone 2g qd and ciprofloxacin 400 mg q12 h for five weeks. The blood cultures became negative after IV antibiotic therapy.

## 3. Discussion

Native valve infective endocarditis is a relatively rare pathology accounting for 2 to 10 cases per 100,000 people per year [3].

*Staphylococcus aureus is* the most common cause of IE with a nearly 30% occurrence rate, followed by *Streptococcus viridans* at nearly 20%, other streptococci at 17.5%, and enterococci at 10.5%. These organisms account for approximately 80–90% of all cases of IE [4]. Gram-negative bacteria, such as *Escherichia coli,* can cause bacteremia, however, rarely cause IE. *Proteus spp* are Gram-negative bacilli and facultative anaerobes that are part of the *Gammaproteobacteria* bacterial lineage and are often associated with urinary tract infections, not IE [5]. *P. mirabilis* possesses a number of different virulence factors including fimbriae and urease which allow it to evade the host immune system [6, 7]. This high virulence may explain how *P. mirabilis* bacteremia may lead to IE [2].

According to a recent systematic review in 2020, there are only 16 cases of IE secondary to *Proteus spp* [2]. There is also another recent case reporting multivalvular IE [1]. All of these cases were in patients who presented with bacteriuria. Our case demonstrates a patient with a rare causative organism for IE with *P. mirabilis,* and it is the first known presentation without a concurrent UTI or asymptomatic bacteriuria. The clinical cure rate, based on this 2020 systematic review, is noted at 62.5%, and mortality was 43.8% [2]. Our patient was treated with meropenem and ultimately deescalated to ceftriaxone and ciprofloxacin. Repeat blood cultures returned negative four days after initiating antibiotic therapy and remained negative. The patient was successfully discharged on IV antibiotics for five more weeks.

There are case reports about systemic infection related to AICD placement. The risks of systemic infections due to AICD cannot be overlooked in this case. This risk ranges from 1.7–4% in 6 months to 2 years, respectively. These cases are more related to subcutaneous infection rather than endocarditis. AICD leads are located in the right atrium and ventricle of the heart. This patient's infection is likely unrelated to AICD as he had no evidence of subcutaneous infection and simply by location of the vegetation in the aortic valve. Additionally, there was no evidence of the vegetation on the AICD leads.

## 4. Conclusion

We report a rare case of IE secondary to *P. mirabilis* infection without a concurrent UTI or asymptomatic bacteriuria. This is the first known case of *P. mirabilis* IE without a urinary tract infection. The mortality rate based on a previous systematic review is reported as high as in these patients. Our patient was able to be treated with dual antibiotic therapy and discharged on the regimen with negative blood cultures.

## Data Availability

The health record data used to support the findings of this case report are restricted in order to protect patient privacy. Where appropriate, certain health record data are included verbatim within the article. This case report also provides a discussion on *Proteus spp*. The data used in the discussion were found within peer-reviewed journals and previously published case reports. Appropriate citations and references are included within the article.
